# Description of changes in clinical outcomes following the implementation of an antibiotic stewardship program in a level IV hospital

**DOI:** 10.7705/biomedica.6748

**Published:** 2023-06-30

**Authors:** Raúl Eduardo Reyes, María José López, Jairo Enrique Pérez, Gustavo Martínez

**Affiliations:** 1 Servicio de Medicina Interna, Hospital Militar Central, Bogotá, D.C., Colombia Servicio de Medicina Interna Hospital Militar Central Bogotá, D.C. Colombia; 2 Servicio de Infectología, Hospital Militar Central, Bogotá, D.C., Colombia Servicio de Infectología Hospital Militar Central Bogotá, D.C. Colombia; 3 Servicio de Infectología, Fundación Cardioinfantil, Bogotá, D.C., Colombia Servicio de Infectología Fundación Cardioinfantil Bogotá, D.C. Colombia

**Keywords:** Antimicrobial stewardship, mortality, hospitalization, antibacterial agents, programas de optimización del uso de los antimicrobianos, mortalidad, hospitalización, antibacterianos

## Abstract

**Introduction.:**

Inadequate prescription of antibiotics has been recognized as a public health problem by the World Health Organization. In this context, antibiotic stewardship programs have been implemented as a tool to mitigate its impact.

**Objective.:**

To describe the changes in clinical outcomes after the implementation of an antibiotic stewardship program in a level IV hospital.

**Materials and methods.:**

We conducted a unique cohort study of patients hospitalized for infectious pathologies that were treated with antibiotics in an advanced medical facility. We collected the clinical history before the implementation of the antibiotic stewardship program (2013 to 2015) and then we compared it to the records from 2018 to 2019 collected after the implementation of the program. We evaluated changes in clinical outcomes such as overall mortality, and hospital stay, among others.

**Results.:**

We analyzed 1,066 patients: 266 from the preimplementation group and 800 from the post-implementation group. The average age was 59.2 years and 62% of the population was male. Statistically significant differences were found in overall mortality (29% vs 15%; p<0.001), mortality due to infectious causes (25% vs 9%; p<0.001), and average hospital stay (45 days vs 21 days; p<0.001); we also observed a tendency to decrease hospital re- admission at 30 days for infectious causes (14% vs 10%; p=0.085).

**Conclusions.:**

The antibiotic stewardship program implemented was associated with a decrease in overall mortality and mortality due to infectious causes, as well as in average hospital stay. Our results evidenced the importance of interventions aimed at mitigating the impact of inadequate prescription of antibiotics.

## Resumen

**Introducción.:**

La inadecuada prescripción de antibióticos es un problema de salud pública, reconocido por la Organización Mundial de la Salud. Los programas de gestión de antibióticos son implementados como una herramienta para mitigar su impacto.

**Objetivo.:**

Describir los cambios observados en los desenlaces clínicos después de la implementación de un programa de gestión de antibióticos en un hospital de IV nivel de atención.

**Materiales y métodos.:**

Se llevó a cabo un estudio de cohorte única de pacientes hospitalizados por patologías infecciosas y tratados con antibióticos en una institución médica de alta complejidad. Inicialmente, se recolectaron las historias clínicas anteriores a la implementación del programa de gestión de antibióticos (2013 a 2015) y luego se compararon con los datos obtenidos después de la implementación del programa de gestión de antibióticos de 2018 a 2019. Se evaluaron los cambios en los desenlaces clínicos como mortalidad y estancia hospitalaria, entre otros.

**Resultados.:**

Se analizaron las historias clínicas de 1.066 pacientes: 266 con historia previa a la implementación del programa y 800 con historia posterior a la implementación. El promedio de edad fue 59,2 años y 62 % de la población era masculina. Se encontraron diferencias estadísticamente significativas en mortalidad global (29 *Vs*. 15 %; p<0,001), mortalidad por causa infecciosa (25 % *Vs*. 9 %; p<0,001) y promedio de estancia hospitalaria (45 *Vs*.21 días; p<0,001), con tendencia a disminuir nuevas hospitalizaciones en 30 días por patología infecciosa (14 *Vs*.10 %; p=0,085)

**Conclusiones.:**

El desarrollo del programa de gestión de antibióticos se asoció con a una disminución en la mortalidad global, la mortalidad por causa infecciosa y la estancia hospitalaria. Esto demuestra la importancia de desarrollar intervenciones dirigidas a mitigar el impacto de la prescripción inadecuada de antibióticos.

The inappropriate use of antimicrobial therapy is a problem currently recognized by the World Health Organization and other control institutions ^1^^,^^2^. The increase in inadequate prescriptions of antibiotics has proven to have an impact on clinical and microbiological outcomes in healthcare institutions, such as the increase in bacterial resistance and nosocomial infections, increased health costs, and longer hospital stays, among others. These conjoin with the limited therapeutic arsenal currently available and the lack of other control tools ^3^^-^^5^ that simultaneously stimulate the search for strategies to mitigate its impact.

In this context, antimicrobial stewardship programs represent a structured solution in charge of multidisciplinary groups that include a set of actions, such as audit and feedback on antibiotic prescription, education guidelines, restriction on prescribing certain drugs, or pre-approval strategies, among others. These interventions mitigate the harms of the inadequate prescription of antibiotics ^6^^,^^7^ and impact microbiological outcomes as evidenced in the medical literature, although the clinical outcome landscape lacks solid scientific support ^3^^,^^4^.

In Colombia, several studies have assessed the effects of antimicrobial stewardship programs in terms of restricting the use of certain groups of antibiotics based on the local epidemiology, hospital stay, cost reductions, and the incidence of multidrug-resistant bacteria isolates. These programs encourage results and have no negative effects on patients’ evolution. However, none of them has focused specifically on evaluating the impact on clinical outcomes ^8^^-^^10^.

In this sense, our study aimed to describe the change in clinical outcomes after the implementation of an antimicrobial stewardship program in a level IV hospital.

## Materials and methods

We conducted a single cohort study with addressed patients before and after the antimicrobial stewardship program implementation. The program intervention included a prospective post-audit model in charge of a surveillance committee of specialists in infectious diseases, internal medicine residents, and nursing groups. This committee used a database of antibiotic initiation formats focused on broad-spectrum antibiotics and did not include restricted drugs. The committee also consulted clinical histories and held counseling with the medical staff providing feedback on antibiotic prescriptions and promoting educational awareness campaigns on the rational use of these drugs, including information on the institutional guidelines for infectious diseases.

In cases where the inadequate prescription was detected, we consulted with the Infectiology Department to select the appropriate medication, dosing, and administration pathway. Additionally, the antimicrobial stewardship group programmed visits to the intensive care unit and the emergency department to review specific cases and check the proper filling out of the initiation format.

We included patients over 18 years of age, diagnosed with infectious diseases requiring antibiotic treatment, and enrolled in the institution’s antimicrobial stewardship program database, from January 2018 to January 2019. We did not use data from 2016 and 2017 considering this a prudential time to consolidate the implementation and stabilization of the program.

We selected a control group from the hospital’s microbiology database, which included patients from 2013 to 2015, before the implementation of the antimicrobial stewardship program. Patients who died 72 hours after hospital admission were excluded as the time was insufficient to evaluate the effect of the program interventions on clinical outcomes. We also excluded those records that did not have all the clinical variables of interest.

We evaluated the following clinical outcomes: overall mortality, mortality due to infectious causes (grouped among the institution’s top ten causes of morbidity due to infection), hospital stay length (days), hospital readmission due to infectious causes in the 30 days following discharge, and average duration of antibiotic therapy.

Urinary infection was defined as urinary tract symptoms with positive microbiological isolation from urine culture. Pneumonia was defined by new or persistent radiographic features with no other obvious cause and two of the following findings: leukocytosis or leukopenia, cough or sputum production, fever or hypothermia, abnormal lung examination, or increase of oxygen requirements. Sepsis of unknown origin was considered in patients with organic dysfunction defined by a Sequential Organ Failure Assessment (SOFA) score greater than two points, secondary to a presumably infectious cause without an evident focus of infection after a rationale clinical study. Bacteremia was diagnosed with a positive blood test culture associated with sepsis without potential infectious foci.

The antimicrobial stewardship program active surveillance also considered the population’s sociodemographic characteristics, the most frequent pathologies with the wrong prescription of antibiotics, and the proportion of patients whose antimicrobial therapy was adjusted as part of the program. The research ethics committee of the *Hospital Militar Central* approved the study.

Considering a minimum of 252 in the before-implementation program group and a 774 after-implementation program group, we calculated a proportion difference of 7%, a sample size ratio of 3, a confidence level of 95%, and a power of 90%.

First, we described the obtained data in terms of frequencies and percentages for the qualitative variables. For the quantitative variables, data distribution was expressed as mean and standard deviation or median and interquartile range.

We analyzed two groups of patients: from 2013 to 2015 (before the antimicrobial stewardship program) and from 2018 to 2019 (after antimicrobial stewardship program) to evaluate potential differences between them (p<0.05 was considered significant) (table 1). All clinical outcomes before and after the program implementation were evaluated (table 2), and an exploratory analysis of mortality due to infectious causes before and after the program was also performed and grouped into the five most prevalent etiologies (table 3).

**Table 1:** General characteristics of the population.

Characteristics	Total population N=1,063	Pre-ASP n=266	Post-ASP n=797	p
Age, years (X; SD)	59.2; 22.7	58.1; 22.6	59.5; 22.7	0.387
Males, [n (%)]	659 (62)	153 (58)	506 (63)	0.131
lnfectious disease	n (%)	n (%)	n (%)	p
Urinary tract infection	322 (30)	83 (31)	239 (30)	0.657
Pneumonia	174 (16)	46 (17)	128 (16)	0.604
Operative site infection	53 (5)	22 (8)	31 (4)	0.04
Bacteremia	100 (9)	25 (9)	75 (9)	0.977
lnvasive fungal infection	5 (0.4)	1 (0.1)	4 (1)	0.8
Soft tissue infection	88 (8)	18 (7)	70 (9)	0.316
Osteomyelitis	42 (4)	0 (0)	42 (5)	< 0.001
Gut-origin sepsis	73 (7)	21 (8)	52 (7)	0.426
Febrile neutropenia	49 (5)	18 (7)	31 (4)	0.049
Neuroinfection	17 (2)	9 (3)	8 (1)	0.007
Superinfected ulcers	15 (1)	1 (0)	14 (2)	0.1
Exacerbated chronic obstructive pulmonary disease	10 (1)	0 (0)	10 (1)	0.067
Sepsis of unknown origin	115 (11)	22 (8)	93 (12)	0.131
Antibiotic treatment				
Duration of antibiotic therapy, days (X; SD)	10.4; 8.3	12.5; 7.7	9.7; 8.4	< 0.001
ASP-modified antibiotic therapy, [n (%)]	--	--	187 (23)	NA

ASP: antibiotic stewardship program

**Table 2:** Clinical outcomes before and after implementation of the antimicrobial stewardship program

Clinical outcome	Total population N=1,063	Pre-ASP n=266	Post-ASP n=797	p
Overall mortality [n (%)]	195 (18)	78 (29)	117 (15)	< 0.001
Mortality due to infectious diseases [n (%)]	136 (13)	65 (25)	71 (9)	< 0.001
Length of hospital stay, days (range)	43.1 (24) (1 - 959)	77.8 (45) (1 - 959)	31.6 (21) (2 - 693)	< 0.001 0.085
Re-entry after 30 days due to infection, [n (%)]	118 (11)	37 (14)	81 (10)	

**Table 3:** Pre-ASP and post-ASP mortality due to infectious causes

Infectious disease	Pre-ASP (n=78) n (%)	Post-ASP (n=117) n (%)	p
Urinary tract infection	16 (21)	7 (6)	< 0.001
Pneumonia	18 (27)	19 (14)	0.001
Bacteremia	5 (7)	11 (14)	0.529
Gut-origin sepsis	4 (6)	10 (19)	0.986
Soft tissue infection	5 (8)	2 (3)	< 0.001

ASP: antimicrobial stewardsthip program

Numerical differences were explored with a bivariate analysis to look for differences between proportions. Quantitative variables were cross-checked with Student’s t test when they presented a normal distribution, and with the Mann-Whitney U test when they did not. The most frequent infectious pathologies requiring the prescription of antibiotic therapy were described and analyzed according to the original strata and expressed as total values and percentages. The hospital’s group of specialists on infectious diseases also described the relationship between infections and inadequate antibiotic prescription regarding the relevance, dosage, and route of administration (table 4).

**Table 4:** Diseases related to inadequate prescription of antibiotics

Infectious disease	Cases (n)	Inappropriate prescription (n)	%
Urinary tract infection	241	49	20.3
Pneumonia	128	18	14.1
Sepsis of unknown origin	93	28	30.0
Bacteremia	75	12	16.0
Soft tissue infection	70	12	17.1
Gut-origin sepsis	52	6	11.5
Osteomyelitis/septic arthritis	42	1	2.4
Operative site infection	31	0	0.0
Febrile neutropenia	31	2	6.4
Superinfected ulcer	14	4	28.5
Exacerbated chronic obstructive pulmonary disease	10	2	20.0
Neuroinfection	8	1	12.5
Invasive fungal infection	5	0	0.0

## Results

**Figure 1:**
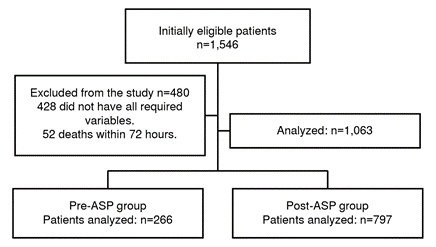
Selection of candidates for the study

We compiled information on 1,546 patients. Of these, we excluded 483 from the analysis: 431 because all the clinical variables of interest were not registered, and 52 died within 72 hours of hospital admission. We finally included 1,063 patients in the study: 266 in the before-implementation program group and 800 after its implementation (figure 1).

No statistical differences were found regarding the age of both groups (p=0.387). The average age of the sample was 59.2 years and 62% was male. The distribution of the infectious etiology was similar in both groups except for osteomyelitis and febrile neutropenia, which were less frequent in the group before the program implementation. The most frequent etiology was urinary tract infection (30%), followed by pneumonia (16%), and sepsis of unknown origin (11%). A statistically significant reduction in the average duration of antibiotic therapy was found after the program implementation (12.49 days vs. 9.74 days; p< 0.001) with a total difference of 2.75 days between the two groups.

As for the clinical outcomes evaluated, there were statistically significant differences after the implementation of the antimicrobial stewardship program in overall mortality (29% vs. 15%; p<0.001), mortality due to infectious causes (25% vs. 9%; p<0.001), and average hospital stay (45 days vs. 21 days; p<0.001) with a tendency to decreasing hospital re-admission after 30 days due to infection (14% vs.10%; p=0.085).

The analysis of mortality due to infectious causes, classified among the five most prevalent etiologies in the hospital, showed a statistically significant difference in the after-implementation group mortality in urinary tract infection (21% vs. 6%; p<0.001), pneumonia (27% vs. 14%; p<0.001), and soft tissue sepsis (8% vs. 3%; p< 0.001), but no statistically significant difference in mortality due to bacteremia (7% vs. 14%; p=0.529) and gut-origin sepsis (6% vs. 19%; p=0.986).

The infectious pathologies mostly associated with an inadequate antibiotic prescription, defined by the antimicrobial stewardship program surveillance committee, were superinfected ulcers (28.5%), urinary tract infection (20.3%), and pneumonia (14.1%).

As a result of the antimicrobial stewardship program active surveillance, antibiotic therapy (suspension or drug change) was modified in 23% of the cases (187/800 patients) in the after-implementation program group.

## Discussion

The conduction of this study addresses inadequate antibiotic prescription, a public health problem that has grown exponentially and requires a measurable impact mainly on microbiological results but also on clinical outcomes ^3^^,^^11^^-^^13^. The benefits of antimicrobial stewardship programs, in terms of clinical indicators, remain a challenge since there is divergent evidence that does not allow reliable conclusions ^14^.

In the present study, we reduced the average duration of antibiotic therapy by 2.75 days. According to previous studies, this translates into cost reduction, lower antibiotic use, and lower bacterial resistance selection pressure because of reduced antibiotic exposure ^15^. Additionally, the active surveillance process on the antimicrobial stewardship program allowed modifications of the antibiotic therapy, according to the recommendations by the infectious disease group, in approximately one out of four patients, which probably had an impact on the clinical outcomes obtained.

The main reasons for therapy modification were an inadequate prescription, treatment adjustment to the microorganisms isolated from cultures, dosage or incorrect route of administration. These factors were discussed in educational sessions in our institution as part of antimicrobial stewardship program feedback. The population characterization allowed us to identify the most prevalent infectious pathologies needing antibiotic treatment and those with a higher proportion of cases receiving an inadequate prescription. These data are relevant to consider in subsequent analyses.

Considering that 25 to 40% of hospitalized patients received antibiotic therapy as part of their treatment, our results will help improve and adjust the medical personnel’s training processes and awareness as part of the derived plan from the antimicrobial stewardship program ^16^. In our study, 16.8% of the patients had an inadequate antibiotic prescription to treat superinfected ulcers, urinary tract infections, and soft tissue infections, very similar to the findings of other studies ^13^.

Regarding the observed mortality, discriminated by infection, it is known that adherence / the accomplishment of antimicrobial stewardship programs represents a reduction in mortality in specific clinical scenarios ^17^^,^^18^. Our study showed a reduction in mortality from urinary tract infection, pneumonia, and soft tissue sepsis after the antimicrobial stewardship program implementation. These can be explained to some extent as the effect of the program’s active surveillance. In the case of bacteremia and gut-origin sepsis, we did not find statistically significant differences, probably because these clinical outcomes are affected by factors other than antibiotic medication, such as the complexity of these conditions, the use of medical devices like intravascular catheters, and the importance of quality care in intra-abdominal surgical pathologies including post-surgical infection control.

The impact of the antimicrobial stewardship program implementation on the different clinical outcomes yielded diverging results due to some methodological limitations ^3^. The meta-analysis published by Agents *et al.* included a large number of studies about antibiotic stewardship programs and assessed their impact in terms of clinical and economic outcomes. The research reported decreases in total antibiotic consumption, length of hospital stays, use of restricted antibiotics, and nosocomial infections by some multidrug-resistant bacteria. However, overall mortality and mortality due to infection were not significantly different after the program implementation. The authors concluded that studies with longer follow-up periods and antimicrobial stewardship standardization are required to draw more precise conclusions ^14^. Likewise, the systematic review by Kaki *et al.* claimed that the absence of studies with better methodological quality is the main limitation in determining the impact of this type of program on clinical outcomes. Mortality and length of hospital stay did not adversely vary with the introduction of the antimicrobial stewardship program ^3^.

Regarding the study’s limitations, we acknowledge an absence of standardization in the clinical variables of interest that forced us to discard a significant group of patients registered in the database, which prevented a more precise analysis of the results. The average age of the analyzed population (59.3 years) implies higher overall mortality rates, comorbidity rates, and a higher probability of adverse effects related to the prescription of antibiotics, which can limit results interpretation. The study’s hospital is the healthcare referral center for all military forces in the country, which can impact on the complexity of the observed clinical cases as reflected in the high mortality related to most of the infectious etiologies evaluated.

Despite the observational type of the study and its potential risk of inherent bias, our data suggest that antimicrobial stewardship program implementation favorably impacts the evaluated clinical outcomes, such as overall mortality, mortality due to infectious causes, length of hospital stay, and average duration of antibiotic therapy. There was also a quantitatively though not statistically significant difference in the reduction of hospital readmission for infection.

However, these results should not be explained exclusively by the program implementation since the strategy was accompanied by other interventions that may have influenced the results, such as stricter handwashing policies, systematic visits of infectious disease specialists to the intensive care unit to provide counsel on the best alternatives for antibiotic therapy, training processes and socialization of clinical practice guidelines, and stricter policies on antibiotic prescription processes. These factors should certainly be considered when interpreting these findings.

Our results should be interpreted with caution. We emphasized that the impact on clinical outcomes was probably a consequence of the overall care quality improvement of infected patients in this institution and that the antimicrobial stewardship program was an adjunct tool in this enhancement plan.

The inadequate prescription of antibiotics is a latent threat to public health. Hence, we must intensify the search for tools to mitigate its impact. Antimicrobial stewardship programs are part of the available strategies with scientific support. However, its standardization and generic introduction into health systems should be encouraged to allow the development of studies with bigger sample sizes and better methodological designs to enable precise conclusions on their impact on clinical outcomes.
